# Relative Deprivation and Prosocial Tendencies in Chinese Migrant Children: Testing an Integrated Model of Perceived Social Support and Group Identity

**DOI:** 10.3389/fpsyg.2021.658007

**Published:** 2021-06-08

**Authors:** Meng Xiong, Lei Xiao, Yiduo Ye

**Affiliations:** ^1^School of Education and Sports Sciences, Yangtze University, Jingzhou, China; ^2^Department of Psychology, University of Edinburgh, Edinburgh, United Kingdom; ^3^School of Psychology, Fujian Normal University, Fuzhou, China

**Keywords:** relative deprivation, prosocial tendencies, perceived social support, in-group identity, migrant children

## Abstract

As a particularly vulnerable group, children from rural areas in China whose families migrate to urban areas often encounter social exclusion, prejudice, and discrimination as they adjust to city life. Hence, migrant children may experience a sense of relative deprivation when they feel they are treated unjustly when compared to their urban counterparts. Although previous research has demonstrated that relative deprivation is a risk factor for prosocial tendencies, this association has not yet been examined in the population of migrant children in China. Further, few studies have revealed the mediating and moderating mechanisms between relative deprivation and prosocial tendencies. Therefore, this study constructed an integrated model examining the possible mediating role of perceived social support and moderating role of in-group identity on the association between relative deprivation and prosocial tendencies. A large sample of 1,630 Chinese rural-to-urban migrant children (845 girls; *M*_age_ = 12.30, *SD* = 1.74) completed a battery of self-report questionnaires regarding relative deprivation, prosocial tendencies, perceived social support, in-group identity, and demographic variables. The results indicated that relative deprivation was negatively correlated with migrant children's prosocial tendencies and this connection was partially mediated by perceived social support. Moderated mediation analysis further indicated that in-group identity moderated the effect of perceived social support on prosocial tendencies, with a high level of in-group identity strengthening the positive association between perceived social support and prosocial tendencies. Parents, educators, and other members of society concerned about migrant children's psychosocial adaptation should provide adequate social support resources and help them foster positive in-group identity to migrant populations to mitigate the adverse effects of relative deprivation and promote their prosocial tendencies.

## Introduction

With the accelerated development of the urbanization process in China, rural-to-urban migration has gradually become one of the most salient contextual factors shaping Chinese family life in the twenty-first century (Wang and Mesman, 2015). Based on a recent report, 35.8 million rural-to-urban migrant children currently live in metropolitan cities in China (National Health Planning Commission, 2017). Research shows that the preservation of the current *hukou* (required governmental registration of all individuals and families living in a particular area in China) system might extend unfair treatment to rural-to-urban migrants and be little the migrants as a disadvantaged social group (Kuang and Liu, 2012). Therefore, it is easy to induce subjective relative deprivation in migrant children's minds due to the loss of benefits that they think they deserve. Furthermore, considerable evidence has indicated that perceived status-based discrimination can increase emotional-behavioral problems in migrant children (Lan et al., 2020).

Rural-to-urban migrants usually work on so-called “3D jobs” (dangerous, dirty, and demeaning; Kuang and Liu, 2012). Due to this, they often face substantial economic pressures (Hernandez et al., 2007). According to the family stress model, low economic resources pose a risk for problems in child development through processes of maladaptive childrearing by stressed parents who lack the resources to provide warm and supportive care (Conger and Donnellan, 2007). Therefore, the perceived social support of migrant children is generally lower than that of urban children. Moreover, permanent urban residents have little desire for contact with the migrants unless it is essential (Lu, 2006). Social distance between the two groups has thus gradually increased (Kuang and Liu, 2012).

Additionally, it is known that relative deprivation often leads to anger, frustration, and low commitment to social norms (Crosby, 1976; Bernburg et al., 2009; Smith et al., 2012). Thus, the probability of these children engaging in prosocial behaviors/tendencies—such as donating, volunteering, and helping others—is limited. Empirical evidence demonstrates that perceived social support is not only an important psychological resource for individuals to cope with stress but also has great significance for understanding and predicting individual psychosocial adjustment (Brissette et al., 2002; Ye, 2005). Previous research has also indicated that group identity plays a moderating role in the relationship between intrinsic and extrinsic motivation and people's compliance with societal norms (Barreto and Ellemers, 2000). Considering the critical role of prosocial development in traditional Chinese culture, it is potentially meaningful to regard prosocial tendencies as the study outcome in the present research (Lan and Wang, 2020). In summary, the aim of the present study is to construct an integrated model, namely a moderated mediation model, to assess the possible mediating role of perceived social support and moderating role of in-group identity between relative deprivation and prosocial tendencies in Chinese migrant children. The strengths of this study lie in focusing on a vulnerable under-researched population, namely, rural-to-urban migrant children in China. The model tested may also be of some value: though the relationships between the variables of the study have been previously investigated, this study proposes an integrated model that combines them.

## Theory and Hypotheses

### Relative Deprivation and Prosocial Tendencies

Relative deprivation refers to a kind of subjective cognition and affective experience in which individuals or groups perceive that they are in a disadvantaged position in horizontal or vertical comparisons with reference individuals or groups, coupled with the emergence of negative emotions such as anger and resentment (Crosby, 1976; Smith et al., 2012; Xiong and Ye, 2016). According to the equity theory (Adams, 1963), whether people are satisfied with their own rewards depends not on the absolute values of their actual rewards, but on the relative values of social comparison with others or historical comparison with themselves. Individuals may believe that they have been treated unfairly, and such a sense of unfairness is closely and negatively associated with prosocial tendencies.

Prosocial tendency is typically defined as voluntary behavior/tendency intended to benefit others (Eisenberg, 2006). Empirical research shows that children's prosocial tendency is an important factor that promotes their social development and personality formation (Miller et al., 1996). Further, prosocial tendency is conducive to a positive interaction between migrant and urban children to better integrate migrant children into the city (Kuang and Tan, 2019). However, numerous studies have shown that the levels of prosocial tendencies of migrant children are significantly lower than those of urban children (Li and Liu, 2013; Kuang and Tan, 2019). Therefore, the cultivation of migrant children's prosocial tendencies is an issue that both the state and individuals should pay attention to.

A previous study has shown that infants' expectations of fair distribution may be significantly related to prosocial behavior/tendency such as empathy, helping others, and sharing based on their sensitivity to others' internal states (Sommerville et al., 2013). Therefore, individuals with a sense of relative deprivation may perceive that they have been deprived of their rights by others, which may induce a sense of unfairness, which, in turn, may inhibit their prosocial tendencies and lead them to regard themselves as victims of unfair treatment (Runciman, 1967; Crosby, 1976; Smith et al., 2012). As a result, these people may not help others because they think of themselves as the ones needing help. Numerous studies have also suggested that individual-based relative deprivation decreases prosocial behavior/tendency (Xiong, 2015; Zhang et al., 2016). Therefore, the first hypothesis of this study is that higher levels of relative deprivation are associated with lower prosocial tendencies among migrant children (H1).

### Perceived Social Support as a Mediator

Perceived social support, which refers to the expectation and evaluation of social support and the belief of possible social support, is an important concept in the study of the structural components of social support (Barrera, 1986; Dunkel-Schetter, 1990). Previous research has shown that psychological maladjustment in children from divorced families is one of the factors most commonly associated with the lack of social support, especially perceived social support (Wang and Yu, 2005). Similarly, as children migrate from rural areas to urban regions, there is also a process of psychological adjustment that may be influenced by social support (Guo et al., 2005; Xiong and Ye, 2011). The core of relative deprivation is the process of social comparison (Appelgryn and Bornman, 1996; Stiles et al., 2000; Zhang et al., 2011), in which the relatively vulnerable individuals may have a perception of relative deprivation when comparing themselves to advantaged others; this perception tends to produce negative emotions such as anger, dissatisfaction, and hatred, which, in turn, may weaken their perception of support from family, friends, and society (Smith and Pettigrew, 2014). Empirical evidence also shows that higher levels of social support are associated with lower levels of relative deprivation, indicating that social support is an essential protective factor for alleviating the adverse impact of relative deprivation on individual development (Zhang and Tao, 2013; Han et al., 2017; Zhang and Liu, 2019).

When people have close and stable social relations, they are more likely to feel cared for by others and have a higher sense of security, which, in turn, makes them more generous and helpful (Twenge et al., 2007). Research on the relationship between social support and prosocial tendencies also demonstrates that individuals with higher levels of social support may exhibit more prosocial tendencies (Gest et al., 2001; Calvete et al., 2010). According to the social support differentiation model (Barrera, 1988; Smith et al., 2012), some stress events, especially traumatic or humiliating events, tend to lead to a decrease in perceived social support, which may then lead to a decrease in individual adjustment levels. Therefore, relative deprivation, a typical stress event, may reduce an individual's prosocial tendencies by reducing their level of perceived social support. Moreover, according to the social support resource theory, as an external protective resource, social support can provide an individual with continuous mental energy that can maintain their physical and mental health and ultimately affect their behavioral responses (Hobfoll et al., 1990). Additionally, empirical studies have confirmed that perceived social support partially mediates the positive association between relative deprivation and psychosocial adjustment (Li et al., 2020; Xiong et al., 2020). Therefore, considering that relative deprivation is negatively correlated with perceived social support, which is positively correlated with prosocial tendencies, we speculated that perceived social support might mediate the relationship between relative deprivation and prosocial tendencies in migrant children (H2).

### Group Identity as a Moderator

Group identity, which originates from social identity theory, refers to the psychological connections between individuals and groups based on the meaning of group membership; that is, the degree to which group membership is integrated into individual self-concept (Tropp and Wright, 2001). In contemporary Chinese society, children of internal migrants are less likely to be enrolled in public schools compared to their local urban peers, and even less likely than children who still live in their place of origin (Chen and Feng, 2019). Many migrant children are thus denied entry into urban public schools and are forced to enroll in so-called “migrant children schools,” which are usually small, lack qualified teachers, and do not have standard teaching materials and sanitation services (Wu et al., 2011). Due to this, the companions of migrant children at school are generally other migrant children with whom they gradually form a group. Moreover, previous studies have shown that migrant youth are exposed to negative stereotypes, social isolation, and integration difficulties, which leads to dissatisfaction of their needs for a sense of belonging (Lan and Moscardino, 2021). Therefore, the in-group identification of migrant children plays a very important role in meeting their belongingness needs.

When people belong to a particular group with which they identify, they are more likely to trust their in-group members; this group identity can promote increased interpersonal trust (Huang and Sun, 2013; Xin et al., 2013). Existing research has found that people who trust others are more likely to engage in altruistic behavior than those who do not trust others (Christian Cadenhead and Richman, 1996). Moreover, considerable evidence also indicates that group identity within the community exerts a positive effect on the willingness to help within-group members (Dovidio et al., 1997; Halloran and Chambers, 2011). According to the social identity theory (Tajfel and Turner, 1986), in a disadvantaged position and/or situation, members of vulnerable groups tend to maintain their positive self-images and psychosocial adaptations by enhancing in-group identification. Hence, when in-group identity is prominent, individuals are more likely to have higher prosocial tendencies toward their in-group members (Hackel et al., 2017).

To some extent, group identity is the basis of social support; under the influence of group identity, individuals tend to provide more support for their in-group members and attribute received help to the social support provided by other in-group members (Haslam et al., 2012). Therefore, the level of in-group identity has an important impact on individuals' social support. Previous research shows that ethnic identity may improve individual prosocial tendencies by promoting cultural values (such as familism and family respect; Knight et al., 2016). Moreover, empirical evidence indicates that the stronger the in-group identity, the weaker the negative impact of relative deprivation on mental health (Schmitt and Maes, 2002). Given that prosocial tendencies and mental health are correlated and mutually predictable in many ways (Nantel-Vivier et al., 2014; Son and Padilla-Walker, 2020; Miles et al., 2021), the present study hypothesizes that in-group identity plays a moderating role between perceived social support and migrant children's prosocial tendencies (H3).

### The Current Study

**Figure 1:**
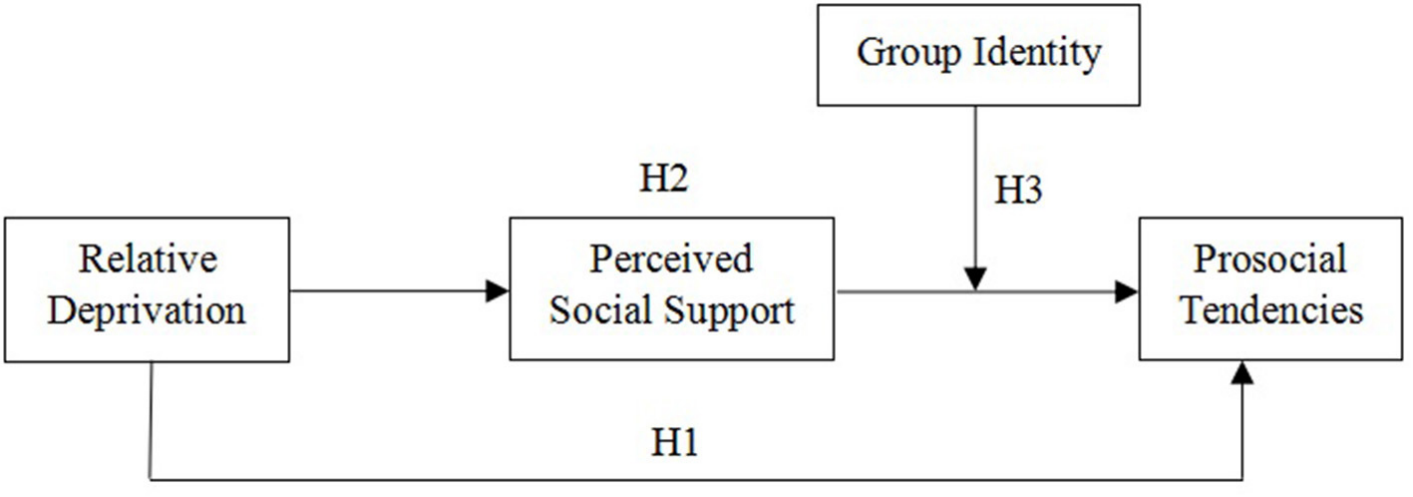
Moderated mediation model of the current study.

Considering the universality of migrant children's relative deprivation and its adverse impacts on individuals' behaviors, it is imperative to examine the mechanisms underlying the link between relative deprivation and prosocial tendencies. To our knowledge, most previous studies have focused on the impact of relative deprivation on undesirable or destructive psychological and behavioral outcomes such as depression, anxiety, aggression, and suicidal ideation (Abrams and Grant, 2012; Smith et al., 2012; Zhang and Tao, 2013), and less on the impact of relative deprivation on positive psychosocial outcomes, such as prosocial tendencies (Turley, 2002; Zoogah, 2010). Given that perceived social support plays a bridging role in the relationship between relative deprivation and depression (Li et al., 2020; Xiong et al., 2020), we examined the mediating effect of perceived social support on the association between relative deprivation and prosocial tendencies. Moreover, previous research has found that in-group identity plays a moderating role in the relationship between intrinsic and extrinsic motivation and people's compliance with norms (Barreto and Ellemers, 2000). Thus, we tested in-group identity as a possible moderator in the relationship between relative deprivation and prosocial tendencies to reveal when the direct and indirect associations between relative deprivation and prosocial tendencies are stronger or weaker. In summary, the current study required the construction of a moderated mediation model (see Figure 1) to test three hypotheses: (H1) relative deprivation is negatively related to migrant children's prosocial tendencies; (H2) perceived social support mediates the relationship between relative deprivation and prosocial tendencies; and (H3) in-group identity moderates the mediating effect of perceived social support in the relationship between relative deprivation and prosocial tendencies. Specifically, in-group identity moderates the second stage of the mediation process (i.e., the link between perceived social support and prosocial tendencies).

## Materials and Methods

### Participants and Procedures

In the present study, Chinese rural-to-urban migrant children were sampled from three coastal cities in southeast China: Fuzhou, Xiamen, and Quanzhou. From each city, we adopted the convenience sampling method to select target schools, namely, one primary school and one junior high school. In the target schools, we used a cluster random sampling method to choose 4th−6th graders from primary school and 7th−9th graders from junior high school. The eligibility criteria for migrant children included the following: (1) there must be no *hukou* in the urban city, and (2) the children must be living with parents who had migrated to the city for employment, more than 6 months ago (Chen et al., 2014). Questionnaire surveys in a paper-and-pencil format were conducted in different classrooms during a class of 30 min. In each classroom, two trained psychology graduate students administered the surveys, answered questions, and monitored the participants' progress. A total of 2,200 questionnaires for migrant children were distributed and 1,993 were returned, with a recovery rate of 90.6%. After verification, 363 invalid responses were eliminated, and 1,630 valid answers were obtained, with an effectivity rate of 81.8%. Among the final sample, 845 were girls (51.8%) and 785 were boys (48.2%); 994 (61.0%) were from primary schools and 636 (39.0%) from junior high schools. The mean age of the participants was 12.30 years (*SD* = 1.74), and the range was 10–15 years. There were 246 (15.1%), 899 (55.1%), and 485 (29.8%) participants, whose length of residence in the city was <3 years, between 3 and 8 years, and over 8 years, respectively. In terms of the educational background of migrant children's parents, 357 (21.9%) reported that their fathers had primary school education or below, 758 (46.5%) reported junior high school education, 383(23.5%) reported senior high school education, and 132 (8.1%) reported college education or above. In addition, 574 (35.2%) of their mothers had primary school education or below, 673 (41.3%) had junior high school education, 275 (16.9%) had senior high school education, and 108 (6.6%) had college education or above. Regarding the family economic statuses, 284 (17.4%) of the participants had an average monthly household income <2,000 yuan, 731 (44.9%) between 2,000 and 5,000 yuan, and 615 (37.7%) had more than 5,000 yuan.

This study was approved by the Ethics Committee for Psychological Research at the corresponding author's institution. All participants, as well as their parents and teachers, provided informed consent before the survey. Participants were asked to complete all of the items regarding relative deprivation, prosocial tendencies, perceived social support, and in-group identity and were informed that their participation would be voluntary, and responses would remain confidential.

### Measures

#### Demographic Form

Participants completed a brief demographic form that included background information on their age, gender, education level, length of residence in the city, parental education, and monthly household income.

#### Relative Deprivation

The Relative Deprivation Scale for Migrant Children (Ye and Xiong, 2017) was used to measure migrant children's sense of relative deprivation. This scale consists of 20 items (e.g., “*How do you think of your family economic status when compared with your urban counterparts*?” and “*How satisfied are you with this situation*?”), measuring five aspects of migrant children's current situation (i.e., family economic status, housing conditions, residential stability, development of personal strengths, and parental involvement in education). The 7-point Likert scale items of the cognitive dimension range from 1 (*very good*) to 7 (*very bad*), and the items of the emotional dimension range from 1 (*very satisfied*) to 7 (*extremely unsatisfied*); higher scores indicate higher levels of relative deprivation. This scale has been used in previous studies with good reliability and validity (Ye and Xiong, 2017; Xiong et al., 2020, 2021). The data of this study showed that the fit indexes of the scale were good (comparative fit index [CFI] = 0.92, Tucker-Lewis Index [TLI] = 0.92, χ^2^/degrees of freedom [*df*] = 4.49, standardized root mean square residual [SRMR] = 0.067). In the present study, Cronbach's α for the scale was 0.92.

#### Prosocial Tendencies

The Chinese version of the Prosocial Tendencies Measure (Carlo and Randall, 2002; Kou et al., 2007) was used to assess migrant children's prosocial tendencies. The scale consists of six dimensions that describe people's prosocial tendencies: openness, anonymity, altruism, compliance, emotionality, and urgency (e.g., “*When other people are around, it is easier for me to help needy others*” and “*It is most fulfilling for me when I can comfort someone who is very distressed*”). This instrument has 26 items and uses a 5-point Likert scale ranging from 1 (*does not describe me at all*) to 5 (*describes me very much*); higher scores indicate higher levels of prosocial tendencies. In the current study, Cronbach's α for the scale was 0.94. Structural validity (CFI = 0.99, TLI = 0.98, χ^2^/*df* =1.67, SRMR = 0.035) was in line with the standards of psychometrics.

#### Perceived Social Support

The Chinese version of the Perceived Social Support Scale (Ong and Ward, 2005; Fan et al., 2012) was used to assess two factors related to social support: emotional support (e.g., “*People who visit you to see how you are doing*”) and instrumental support (e.g., “*People who give you some tangible assistance in dealing with any communication or language problems that you might face*”). Participants answered 18 items on a 5-point Likert scale ranging from 1 (*nobody*) to 5 (*many people*). The higher the scores, the more social support migrant children might receive in those cities. In the present study, Cronbach's α for the scale was 0.96. Structural validity (CFI = 0.98, TLI = 0.98, χ^2^/*df* = 4.21, SRMR = 0.038) was in line with the standards of psychometrics.

#### Group Identity

Migrant children's group identities were measured using the Chinese version of the In-group Identification Scale (Phinney, 1992; Liu et al., 2013). The scale includes 12 items, categorized into two dimensions: emotional identity [e.g., “*I am happy that I am a member of the group I belong to (e.g., the group of migrant children)*”] and cognitive identity (e.g., “*I have a clear sense of my group background and what it means for me*”). Each item was rated on a 6-point Likert scale ranging from 1 (*strongly disagree*) to 6 (*strongly agree*). The average score was taken as the index of in-group identity, with higher scores indicating a stronger identification with their inner group (i.e., the group of migrant children). In the current study, Cronbach's α for the scale was 0.90. Structural validity (CFI = 0.97, TLI = 0.96, χ^2^/*df* = 4.80, SRMR = 0.048) was in line with the standards of psychometrics.

### Data Analysis Strategies

Descriptive statistics and correlation analyses were first conducted using the Statistical Package for the Social Sciences (SPSS) version 22.0. Then, we employed the SPSS macro PROCESS, developed by Hayes (2018), to examine the mediation model (using Model 4) and the moderated mediation model (using Model 14). The macro has been widely used in previous studies to test complex models that include both mediator and moderator variables with the bias-corrected percentile bootstrap method (e.g., van Strien et al., 2016; Liu et al., 2018). Moreover, considering that previous studies found age and gender differences in migrant children's relative deprivation (Zhang and Tao, 2013; Xiong and Ye, 2016), we included age and gender as covariates in all analyses.

The potential common method bias effect was examined by using Harman's single factor test for all of the research items (Podsakoff et al., 2003, 2012). The results showed that there were 11 distinct factors with eigenvalues >1, with the largest factor accounting for 14.97% of the total variance, which was less than the threshold level of 40% (Zhou and Long, 2004). Therefore, the common method bias was not obvious in the present study.

## Results

### Preliminary Analyses

The results of the descriptive statistics and correlation analyses are presented in Table 1. Specifically, relative deprivation was negatively correlated with prosocial tendencies (*r* = −0.26, *p* < 0.01), perceived social support (*r* = −0.28, *p* < 0.01), and in-group identity (*r* = −0.32, *p* < 0.01). Perceived social support was positively correlated with prosocial tendencies (*r* = 0.38, *p* < 0.01) and in-group identity (*r* = 0.53, *p* < 0.01). In-group identity was also positively correlated with prosocial tendencies (*r* = 0.51, *p* < 0.01). These results were consistent with our expectations and supported hypothesis H1.

**Table 1:** Descriptive statistics and correlations among core variables.

**Variable**	***Mean***	***SD***	**Age**	**Gender**	**RD**	**PSS**	**GI**	**PT**
Age	12.29	1.67	1.00					
Gender	—	—	0.00	1.00				
RD	3.24	0.95	0.25^**^	−0.02	1.00			
PSS	2.75	0.97	−0.05^*^	−0.00	−0.28^**^	1.00		
GI	4.01	0.97	−0.05^*^	0.03	−0.32^**^	0.53^**^	1.00	
PT	3.48	0.67	−0.06^*^	0.06^*^	−0.26^**^	0.38^**^	0.51^**^	1.00

*N = 1,630. Gender is a virtual variable: 0, female students, 1, male students; RD, relative deprivation; PSS, perceived social support; GI, in-group identity; PT, prosocial tendencies; SD, standard deviation*.

* *p < 0.05*,

** *p < 0.01*.

### Testing for the Mediation Model

As shown in Table 2, after controlling for gender and age, relative deprivation negatively predicted perceived social support (*B* = −0.29, *p* < 0.001), and perceived social support positively predicted prosocial tendencies (*B* = 0.34, *p* < 0.001). The effect of relative deprivation on prosocial tendencies was also significant (*B* = −0.16, *p* < 0.001), which suggests that perceived social support partially mediated the link between relative deprivation and prosocial tendencies (indirect effect = −0.10, *SE* = 0.01, 95% CI [−0.13, −0.08]); the bootstrap 95% confidence interval for the mediating effect of perceived social support did not contain 0. The mediation effect accounted for 62.5% of the total effect. Thus, hypothesis H2 was supported.

Table 2Summary of mediation results.

**Table:** 

		**Model summary**					
***Y***	***X***	***R***	***R*^2^**	***F***	***B***	**SE**	**95%CI**
PT		0.26	0.07	39.42^***^			
	Age				0.01	0.02	[-0.02, 0.03]
	Gender				0.10^*^	0.05	[0.01, 0.20]
	RD				−0.26^***^	0.03	[–0.31,–0.21]
PSS		0.29	0.08	48.03^***^			
	Age				0.02	0.02	[–0.01, 0.04]
	Gender				−0.01	0.05	[–0.10, 0.09]
	RD				−0.29^***^	0.03	[–0.34,–0.25]
PT		0.42	0.18	92.05^***^			
	Age				−0.00	0.01	[–0.03, 0.03]
	Gender				0.11	0.05	[0.02, 0.19]
	RD				−0.16^***^	0.03	[–0.21,–0.11]
	PSS				0.34^***^	0.02	[0.30, 0.38]

**Table:** 

Effect	*B*	Boot SE	Boot LLCI	Boot ULCI
Direct	−0.16	0.02	−0.21	−0.11
Indirect	−0.10	0.01	−0.13	−0.08

*N, 1,630. RD, relative deprivation; PSS, perceived social support; PT, prosocial tendencies. Bootstrap sample size = 5,000. LL, low limit; CI, confidence interval; UL, upper limit*.

* *p < 0.05*,

*** *p < 0.001*.

### Testing for the Moderated Mediation Model

**Figure 2:**
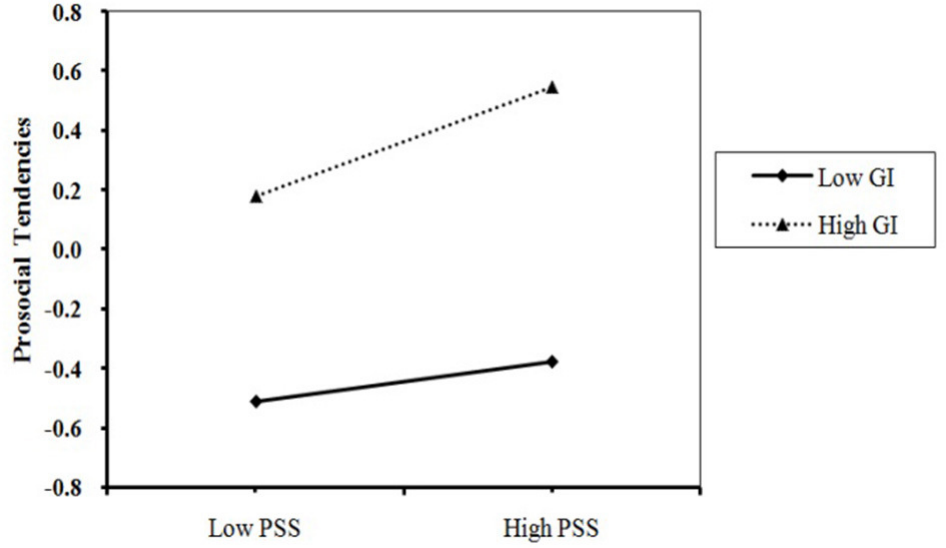
Moderating effect of in-group identity on the prediction of prosocial tendencies.

Table 3 shows the moderating effect of in-group identity in the mediation model after controlling for gender and age. After adding in-group identity as a moderator in the model, the prediction of prosocial tendencies, by the product term of perceived social support and in-group identity, was significant (*B* = 0.07, *p* < 0.001; see Table 3). Furthermore, when performing conditional indirect effect analysis, PROCESS automatically gives the effects of the mediator variable on three levels of the moderator: *M –SD, M*, and *M* + *SD*. As shown in Figure 2, perceived social support had a significant positive predictive effect on prosocial tendencies among participants with higher levels of in-group identity (*simple slope* = 0.20, *t* = 7.27, *p* < 0.001). However, for participants with lower levels of in-group identity, perceived social support had no positive predictive effect on prosocial tendencies (*simple slope* = 0.05, *t* = 1.59, *p* > 0.05), which indicates that the predictive effect of perceived social support on prosocial tendencies decreased with the decline of migrant children's in-group identities. Thus, hypothesis H3 was supported.

Table 3Moderated mediation analysis results with in-group identity as a moderator.

**Table:** 

		**Model summary**					
***Y***	***X***	***R***	***R*^2^**	***F***	***B***	**SE**	**95% CI**
PT		0.54	0.29	113.26^***^			
	Age				−0.01	0.01	[-0.03, 0.02]
	Gender				0.09^*^	0.04	[0.01, 0.18]
	RD				−0.08^***^	0.02	[-0.13,-0.04]
	PSS				0.13^***^	0.03	[0.08, 0.18]
	GI				0.42^***^	0.03	[0.37, 0.47]
	PSS × GI				0.07^***^	0.02	[0.03, 0.10]

**Table:** 

Effect	GI values	*B*	Boot SE	Boot LLCI	Boot ULCI
	M-1SD (-0.97)	−0.018	0.01	−0.044	0.009
Indirect	M (-0.01)	−0.038	0.01	−0.057	−0.020
	M+1SD (1.05)	−0.057	0.01	−0.080	−0.036

*N, 1,630. RD, relative deprivation; PSS, perceived social support; GI, in-group identity; PT, prosocial tendencies. Bootstrap sample size = 5000. LL, low limit; CI, confidence interval; UL, upper limit*.

* *p < 0.05*,

*** *p < 0.001*.

## Discussion

We constructed a moderated mediation model to analyze the psychological mechanisms underlying the relationship between relative deprivation and prosocial tendencies in migrant children. The results showed that perceived social support acted as a mediator, and in-group identity acted as a moderator on the relationship between relative deprivation and prosocial tendencies. Furthermore, the mediating effect of perceived social support was moderated by in-group identity.

### Association Between Relative Deprivation and Prosocial Tendencies

Consistent with previous studies (Zhang et al., 2016; Callan et al., 2017), our findings showed that relative deprivation was negatively correlated with prosocial tendencies. According to the relative deprivation theory (Crosby, 1976; Mummendey et al., 1999; Smith et al., 2012), relative deprivation can lead to emotional experiences of anger and resentment, which may further adversely impact prosocial behaviors/tendencies (Zhang et al., 2016). Anger is a destructive mood state directed at others, often leading to aggressive and antagonistic behaviors (Berkowitz, 1990; Van Coillie and Van Mechelen, 2006; Lemay et al., 2012). Furthermore, anger has been found to inhibit help-giving (Greitemeyer and Rudolph, 2003). Resentment refers to an individual's strong dissatisfaction or hatred that is based on specific reasons (TenHouten, 2006; Sun, 2012). Feelings of resentment and gratitude can be seen as two opposite ends of a bipolar continuum. Moreover, many studies have shown that gratitude promotes prosocial tendencies (Bartlett and DeSteno, 2006; Tsang, 2006; Nowak and Roch, 2007). In contrast, anger and resentment caused by relative deprivation may inhibit prosocial tendencies. Empirical evidence also demonstrates that relative deprivation inhibits prosocial behavior partially through the tendency to prioritize self-interest over others' welfare (Zhang et al., 2016). Therefore, our findings further confirmed that relative deprivation could lead to a decrease in prosocial tendencies.

### Mediating Effect of Perceived Social Support

In the current study, we found that relative deprivation was both directly and indirectly associated with prosocial tendencies through the mediating effect of perceived social support, which was consistent with our expectations. Previous studies have revealed that relative deprivation negatively affects individuals' behaviors (Walker and Smith, 2002; Pettigrew, 2016; Greitemeyer and Sagioglou, 2017). According to the phenomenological variant of ecological systems theory (Spencer et al., 2003), risk contributors (e.g., poverty, racial stereotypes, and racial discrimination) make people susceptible to adverse developmental outcomes. Specifically, as a kind of stress event, relative deprivation may threaten healthy development (Crosby, 1976; Donnenwerth and Cox, 1978; Wright et al., 1999). However, the threat effect of risk contributors may be offset or balanced by support resources and/or perceived social support, thus weakening the negative effect of relative deprivation on prosocial tendencies.

Life changes and stressful events caused by migrating from rural areas to urban regions may expose children to challenges that impact all aspects of their development, such as their parent-child relationships, peer relationships, and social abilities. In turn, these developmental challenges may cause them to perceive themselves as having fewer social support resources (Cummings et al., 2000). Previous research has demonstrated that relative deprivation can also significantly and negatively predict perceived social support (Han et al., 2017). Empirical studies have indicated that social support directly and beneficially affects people's emotional health and overall well-being, as well as plays a positive role in maintaining mental health in high-pressure situations (Wills, 1985; Dean et al., 1990; Reinhardt et al., 2006). Moreover, migrant children's perceived social support from their parents and peers makes them more inclined to help other people in society (Gest et al., 2001; Zhang and Tao, 2013). Therefore, perceived social support may alleviate the negative impacts of relative deprivation on migrant children's prosocial tendencies. From this result, we can suggest that parents, teachers, and other members of society concerned with children's psychosocial adaptation should provide adequate social support resources to migrant children to mitigate the adverse effects of relative deprivation and promote their prosocial tendencies.

### Moderating Effect of Group Identity

This study found that group identity moderates the role of perceived social support in promoting prosocial tendencies in migrant children. This result coincides with the risk and protective factor model (Scal et al., 2003; Xiong et al., 2020), which states that the effect of one risk factor (e.g., relative deprivation) on an outcome (e.g., prosocial tendencies) may be influenced by another protective factor (e.g., group identity). In the current study, migrant children with high in-group identity perceived more social support, which appeared to alleviate the impacts of relative deprivation on their prosocial tendencies.

Specifically, apart from experiencing normal identification and exploration, migrant children may also refine and form group identities related to registered residences while fighting against relative deprivation that may have adverse effects on their development (Fan et al., 2012). If they do not have appropriate support resources and develop appropriate coping strategies, relative deprivation may become a risk factor for increased vulnerability and adverse consequences. Research shows that when an individual identifies with a particular social group, they are more likely to try to help other members of the inner group and are more likely to accept and use the help from other in-group members (Levine et al., 2005). As a result, migrant children with higher in-group identity (i.e., identifying with the group of migrant children) are more willing to trust and accept the help provided by the in-group members (i.e., other migrant children), which may enhance their perceived social support. Furthermore, numerous studies have shown that those who affiliate and identify with their chosen inner group tend to focus on the positive aspects of their inner group that might support and maintain their psychosocial adaptation (Yip et al., 2008; Pascoe and Richman, 2009; Paradies et al., 2015). Previous research has also revealed that when the support provider perceives that a stranger who asks for help holds a social identity similar to their own, the support provider's willingness to help might increase (Levine et al., 2005). In summary, for individuals with high in-group identity, the influence of social support on prosocial tendencies is more significant. Therefore, along with social support resources, parents, educators, and others who are concerned about migrant children's relative deprivation should also foster positive in-group identity, namely identifying with migrant populations, when providing appropriate interventions to improve the children's prosocial tendencies.

### Implications and Limitations

This study has significant theoretical implications. On the one hand, this study deepens what is known from previous research by examining the psychological mechanisms underlying the link between relative deprivation and prosocial tendencies. It contributes to a better understanding of how and when relative deprivation is related to the prosocial tendencies of children who have immigrated to new communities. On the other hand, the results show that relative deprivation is both directly and indirectly associated with prosocial tendencies through the mediating effect of perceived social support. This finding effectively integrates the social support differentiation model (Barrera, 1988; Smith et al., 2012) and social identity theory (Tajfel and Turner, 1986); it also has implications for promoting the development of a more comprehensive model of prosocial tendencies.

There are also several practical implications of this study that should be noted. First, it is necessary for parents and educators to help migrant children develop prosocial tendencies by providing adequate social support. Second, considering that social support is a vital mechanism linking relative deprivation and prosocial tendencies, it will be effective for the education authorities to introduce more supportive policies for migrant children, and for the education executive departments to implement appropriate education policies. For example, migrant children are given equal opportunities to receive high-quality educational resources in cities as urban children. Third, this study suggests that we could reduce the negative impact of relative deprivation by deepening the group identity of migrant children to the inner group. We should actively guide the respect for and recognition of the migrant population in society, and change the public communities' cognition and regard of migrant children.

Despite these theoretical and practical implications, this study has several limitations. First, the results should be interpreted with caution in terms of causality, as the present study collected data using a cross-sectional survey. Future studies should conduct longitudinal or experimental research to confirm causal relationships. Second, the representativeness of the sample may restrict the external validity and generalizability of our findings because our participants were all from one country, namely China. Future research should include participants from diverse countries and/or regions to obtain more robust results. Third, self-report methods may restrict the accuracy of the results due to social desirability and other biases. Future research should collect data from multiple informants. Fourth, considering the multifaced nature of prosocial tendencies and the increasing trend of this research area, future research should possibly unveil the different types (e.g., prosocial tendencies toward in-group and out-group members) and/or dimensions of prosocial tendencies, gaining a more comprehensive picture of the correlations of prosocial tendencies in migrant children. Finally, to avoid ethical risks, this study only uses self-reporting methods to identify migrant children. Further, the study does not distinguish between different types of migrant children, such as those who follow their father, those who follow their mother, and those who follow their parents. Future research should combine subjective reporting with objective criteria and distinguish different types of migrant children, to further validate the findings of this study.

## Conclusion

The focus of most previous studies on relative deprivation has been its effects on undesirable or destructive psychological and behavioral outcomes, neglecting the potential effects of relative deprivation on positive psychosocial outcomes. Further, previous studies have focused less on a vulnerable under-researched group, namely, rural-to-urban migrant children in China. Hence, we constructed an integrated model to fill in the gaps by testing the mediating role of perceived social support and moderating role of in-group identity on the association between relative deprivation and prosocial tendencies in Chinese migrant children. Relative deprivation was significantly negatively correlated with prosocial tendencies, and this connection can be partially mediated by perceived social support. Moreover, in-group identity moderated the effect of perceived social support on prosocial tendencies, with a high level of in-group identity strengthening the positive association between perceived social support and prosocial tendencies. This study provides a new direction for the scientific training of migrant children's prosocial tendencies. Parents, educators, and others who are concerned about migrant children's psychosocial adaptation should provide adequate social support resources and help them foster positive in-group identity to migrant populations to mitigate the adverse effects of relative deprivation and promote their prosocial tendencies.

## Data Availability Statement

The raw data supporting the conclusions of this article will be made available by the authors, without undue reservation.

## Ethics Statement

The studies involving human participants were reviewed and approved by the Ethics Committee for Psychological Research at the corresponding author's institution. The participants and their legal guardian provided their written informed consent to participate in this study.

## Author Contributions

MX conceived and designed the study, performed the survey, and authored and reviewed drafts of the paper. LX analyzed the data, prepared figures and tables, and wrote it into the article. YY conceived and designed the study. All authors were involved in developing, editing, reviewing, and providing feedback for this manuscript and have given approval of the final version to be published.

## Conflict of Interest

The authors declare that the research was conducted in the absence of any commercial or financial relationships that could be construed as a potential conflict of interest.
